# Smoking and 10-year risk of cardiovascular and non-cardiovascular events after contemporary coronary stenting

**DOI:** 10.1016/j.ajpc.2024.100718

**Published:** 2024-08-15

**Authors:** Scott Kinlay, Melissa M. Young, David R. Gagnon

**Affiliations:** a Veterans Affairs Boston Healthcare System, West Roxbury, MA, USA; b Harvard Medical School, Boston, MA, USA; c Department of Biostatistics, Massachusetts Veterans Epidemiology Research & Information Center (MAVERIC) VA Boston Healthcare System, Boston, MA, USA; d Brigham and Women's Hospital, Boston, MA, USA; e Boston University Chobanian & Avedisian School of Medicine, Boston, MA, USA; f Boston University School of Public Health, Boston, MA, USA

**Keywords:** Smoking, Former smoking, Risk, Cardiovascular, Non-cardiovascular, Prevention

## Abstract

The impact of smoking cessation on long-term clinical outcomes after contemporary percutaneous coronary intervention (PCI) is not well known. We estimated the association of smoking and smoking cessation on the 10-year risk of cardiovascular and non-cardiovascular events in patients after contemporary PCI in a multicenter retrospective cohort of all patients having PCI with second generation drug-eluting stents in the VA Healthcare System between 2008 and 2016. Smoking status, comorbidities and clinical outcomes were extracted from the medical record and the National Death Index. Hazard ratios and 95 % confidence intervals (HR, 95 %CI) were derived from Cox Proportional Hazards Models. Estimates of events prevented from smoking cessation were derived from the differences in absolute risks between subjects who smoked and had stopped smoking. Among 29,001 patients, 10,598 (36.5 %) were current smoking patients, 13,093 (45.1 %) were former smoking patients, and 5,310 (18.3 %) never smoked. Over 10 years, 7,806 (26.9 %) subjects died with non-cardiovascular deaths exceeding cardiovascular deaths. In multivariable models, current smoking was significantly associated with increased long-term risks of all-cause death (HR = 1.27, 95 %CI = 1.19, 1.36), myocardial infarction (HR = 1.32, 95 %CI = 1.21–1.43), cancer death (HR = 2.55, 95 % CI = 2.10, 3.08), and pulmonary death (HR = 4.07, 95 % CI = 2.85, 5.83). Smoking cessation may prevent 18.5 % (95 %CI = 16.0 %, 20.9 %) all-cause deaths, 14.8 % (95 %CI = 9.8 %, 19.6 %) cardiovascular deaths, 42.6 % (95 %CI = 37.7 %, 47.2 %) cancer deaths, and 48.3 % (95 %CI = 41.8 %, 54.2 %) pulmonary deaths among smokers. Stopping smoking will likely have major impacts on non-cardiovascular events as well as cardiovascular events in patients after PCI with second generation drug-eluting stents.

## Highlights

• Patients having PCI who smoke are on average 4 years younger than patients who never smoked.
• Smoking is associated with higher risks of cardiovascular *and* non-cardiovascular death after PCI with second generation drug-eluting stents.
• Stopping smoking prevents cardiovascular deaths but may have a greater effect on preventing cancer and pulmonary deaths after PCI with second generation drug-eluting stents.

**Figure:**
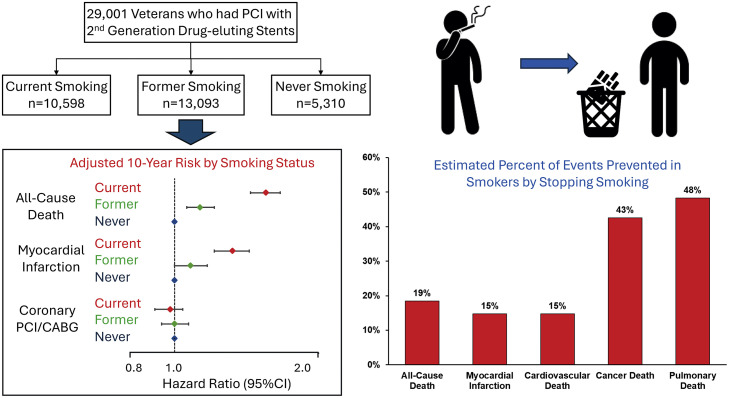
**Central Illustration:** Preventing and stopping smoking have important effects in reducing the risk of cardiovascular and non-cardiovascular events as well as major bleeding after PCI.

## 1 Introduction

Older studies find inconsistent relationships between smoking and adverse cardiovascular outcomes after percutaneous coronary intervention (PCI) [[1], [2], [3], [4], [5], [6], [7], [8], [9], [10], [11], [12], [13], [14]]. These relationships may be confounded by the younger age and lower number of comorbidities with current smoking [1,2,[5], [6], [7],^10^,^11^], or by older age and increased comorbidities associated with former smoking [15]. Many studies combine former smoking with never smoking and may under-estimate the risk of current or former smoking on outcomes [16]. Contemporary risk factor modification and PCI devices have improved cardiovascular outcomes compared to older studies [17], and more powerful antithrombotic therapies may reduce the risk of death and myocardial infarction after PCI [18]. The relationship of smoking to these outcomes and non-cardiovascular death may be different to older studies. We assessed the risk of current smoking and former smoking to adverse cardiovascular and non-cardiovascular outcomes in all patients having PCI in the VA Healthcare System with second-generation drug-eluting stents. We hypothesized that smoking had a similar relationship to the risk of non-cardiovascular events in addition to cardiovascular events in the decade after the PCI.

## 2 Methods

We constructed a retrospective cohort of all patients having PCI with second-generation drug-eluting stents in the VA Healthcare System from 2008 until 2016 from the VA Clinical Assessment Reporting and Tracking (CART) program. [[19], [20], [21]] The CART program records all PCIs in the VA Healthcare System and includes procedural data, such as the dimension and name of stents implanted. These data were imported into the secure VA Informatics and Computing Infrastructure server, and patient data were linked to the VA Corporate Data Warehouse, which provided baseline demographic data, clinical history information, medication prescriptions, and the diagnoses for readmissions. We also linked data on non-VA hospital admission diagnoses from the VA Information Resource Center which links data from the Centers for Medicare and Medicaid Services database (Medicaid inpatient, and Medicare Inpatient files). Death was ascertained from the VA Death Index, and cause of death from the Joint VA and Department of Defense Center of Excellence for Mortality Data Repository linked to the National Death Index. Subjects were excluded if they had missing or incongruent data (e.g. multiple patient identifiers), or missing smoking status. The study was approved by the VA Boston IRB.

Smoking status was extracted from the pre-procedural CART note within 7 days prior to the PCI, which defines current, former, or never smoking in patients based on the proceduralist's interview of the patient. Subjects with earlier classifications of “yes” or “no” were excluded as these did not define former and never smoking. Data from the index PCI included patient sex, race, prior myocardial infarction (MI), prior PCI, prior coronary artery bypass grafting (CABG), prior stroke, prior major bleed, and smoking status. We defined comorbidities based on International Classification of Diseases, 9th revision and 10th revision (ICD-9, ICD-10) in the 5 years before PCI (Table S1). Baseline comorbidities were assessed from 5-years prior to the index PCI, except for prior MI and CABG which were defined as any occurrence up until 15 days prior to the index PCI. An acute coronary syndrome (ACS) at the index PCI was defined from 7-days before until 14-days after the index PCI, based on prior studies showing that events in the first 14 days may represent an ACS leading to the index PCI [22].

Procedural data were extracted from the CART record and included the stent minimum and maximum nominal diameters (smallest and largest diameters of all stents implanted), number of stents (total number used in the procedure), fluoroscopy time and contrast volume used for the case.

### 2.1 Outcomes

Outcomes of death, cause of death, myocardial infarction, and repeat coronary revascularization were assessed after the index PCI. We determined the clinical outcomes starting 14 days after the initial PCI using ICD9 and ICD10 codes (Table S1), as an earlier study in Veterans showed that events within 14 days may relate to salvage cases or technical issues with the index PCI [22]. Cause of death data was available for 81 % (6389/ 7896) of deaths as this data is available from the National Death Index later than all-cause mortality. Cause of death was grouped using ICD10 codes into cardiovascular death (cardiac or cerebrovascular disease), cancer death, pulmonary death (chronic obstructive pulmonary disease or pulmonary infection), other infection death, or other death (Table S2).

### 2.2 Statistics

Clinical outcomes were followed up to a maximum 12 years after the index stent. Baseline demographics, comorbidities, and stent dimensions were described as means and standard deviations (SD) or frequencies and percents as appropriate. Event curves for the outcomes compared subjects in three groups of current smoking, former smoking, and never smoking. The main analysis consisted of examining the risk of the 3 primary outcomes (death, myocardial infarction, repeat coronary revascularization). Secondary outcomes were causes of death grouped as described above. Hazard ratios and 95 % confidence intervals (95 % CI) over 10 years for each clinical outcome were estimated from Cox proportional hazards regression models. Subjects were censored at the end of the follow-up period (2/29/2020), 18 months after the last medical record in the VA electronic medical record, or the date of death (for non-death outcomes). We used multivariable Cox proportional hazards models to adjust for confounders related to the outcome of interest including age, sex, race, ethnicity, acute coronary syndrome at the index PCI, anemia, congestive heart failure, chronic kidney disease, insulin requiring diabetes mellitus, oral hypoglycemics, hypertension, peripheral artery disease, prior myocardial infarction, prior coronary artery bypass grafting, total cholesterol, low-density lipoprotein cholesterol, systolic blood pressure, body mass index, anticoagulants, and statins. We adjusted for DAPT discontinuation by starting a new record for a subject if they discontinued DAPT which was stratified by < 10 months and ≥ to 10 months after the index PCI based on prior analyses [19,21]. Medications and comorbidities were updated at the start of a new record. Chronic obstructive pulmonary disease (COPD) was not used as a covariate in the primary analyses because of collinearity with smoking status and COPD death, but sensitivity analyses included this variable in models (supplementary data). We also assessed models by sex and by the indication for the index PCI (acute coronary syndrome versus no acute coronary syndrome). We used the difference in risk between current smoking versus former smoking to estimate the potential impact of smoking cessation over 10 years in smokers also expressed as the attributable risk in the exposed (percentage of preventable events in smokers) and number needed to treat (NNT) to prevent one event in smokers based on risk estimates from adjusted survival models [23]. We also assessed the risk of myocardial infarction and repeat coronary revascularization in competing risks models accounting for the competing risk of death. All programming used SAS statistical software. As there were 3 major outcomes, we defined statistical significance at the *p* < 0.01 level to account for multiple comparisons.

## 3 Results

**Fig. 1:**
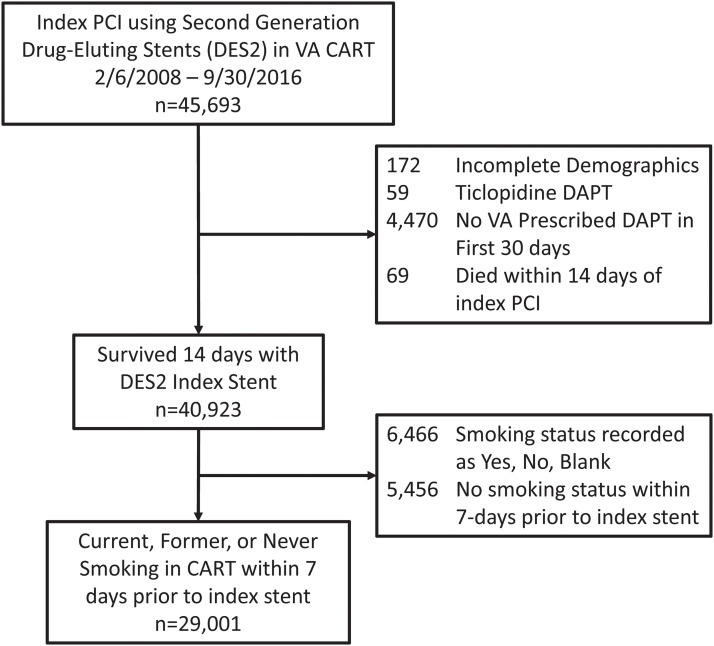
flowchart of inclusions and exclusions.

From 2008–2016, 45,693 unique patients had a PCI with second-generation drug-eluting stents in the VA Healthcare System. Of these, 29,001 had PCI with a second-generation drug-eluting stent, survived at least 14 days after their PCI, and had smoking status recorded (Fig. 1).

Table 1 shows the baseline characteristics of subjects by smoking status at the index PCI. On average, current smoking patients were 4 years younger than never smoking patients and 5 years younger than former smoking patients. Current smoking patients more often identified themselves as Black and less often as Hispanic or Latino, more frequently had an acute coronary syndrome at their index PCI, and more likely to have COPD or peripheral artery disease – comorbidities causally related to cigarette smoking. Current smoking patients had a lower body mass index, and were less likely to have diabetes mellitus, anemia, chronic kidney disease, and prior CABG than never or former smokers – factors related to lower body weight or younger age. Although most patients (>75 %) were on statin therapy and the mean systolic blood pressure was 132–134 mmHg, current smoking patients were slightly less likely to be on statin therapy and more likely to have stopped dual antiplatelet therapy within 10 months of their index PCI, a group we previously defined at higher risk [19,21]. Follow-up ended in February 2020 with patients followed until death or an average 6.5 years and a maximum of 12 years after their index PCI.

**Table 1:** Description of Subjects at the Index PCI.

Baseline Characteristics	Baseline Smoking Status		
	Current *n* = 10,598	Former *n* = 13,093	Never *n* = 5310
Age, years, mean (SD)	61.9 (7.7)	67.1 (8.4)	65.9 (9.5)
Men, n (%)	10,376 (97.9)	12,971 (99.1)	5181 (97.6)
Race			
American Indian or Alaska Native, n (%)	96 (0.9)	99 (0.8)	34 (0.6)
Asian, n (%)	32 (0.3)	55 (0.4)	48 (0.9)
Black or African American, n (%)	1639 (15.5)	1404 (10.7)	674 (12.7)
Native Hawaiian/ Other Pacific Islander, n (%)	72 (0.7)	85 (0.6)	36 (0.7)
White, n (%)	8322 (78.5)	10,773 (82.3)	4250 (80.0)
Unknown or Null, n (%)	437 (4.1)	677 (5.2)	268 (5.0)
Ethnicity			
Hispanic or Latino, n (%)	343 (3.2)	664 (5.1)	354 (6.7)
Not Hispanic or Latino, n (%)	9943 (93.8)	11,987 (91.6)	4779 (90.0)
Unknown or Null, n (%)	312 (2.9)	442 (3.4)	177 (3.3)
Acute Coronary Syndrome at Index PCI, n (%)	6558 (61.9)	7160 (54.7)	3087 (58.1)
Body mass index, kg/m^2^, mean (SD)	29.6 (5.9)	31.3 (5.9)	31.3 (5.8)
Total Cholesterol, mg/dL, mean (SD)	172.3 (45.8)	163.4 (45.0)	168.4 (46.0)
LDL Cholesterol, mg/dL, mean (SD)	100.5 (38.7)	91.8 (36.2)	97.2 (38.3)
Systolic blood pressure, mmHg, mean (SD)	131.6 (19.9)	133.9 (19.7)	134.4 (19.6)
Diastolic blood pressure, mmHg, mean (SD)	76.5 (12.0)	75.2 (11.2)	76.5 (11.5)
**Comorbidities**			
Anemia, n (%)	1611 (15.2)	2911 (22.2)	1082 (20.4)
Congestive heart failure, n (%)	2345 (22.1)	3389 (25.9)	1109 (20.9)
Chronic kidney disease, n (%)	1329 (12.5)	2535 (19.4)	1040 (19.6)
Chronic obstructive airways disease, n (%)	4384 (41.4)	3999 (30.5)	824 (15.5)
Diabetes mellitus			
Requiring insulin, n (%)	3033 (28.6)	5007 (38.2)	1960 (36.9)
Using oral hypoglycemics, n (%)	3511 (33.1)	5762 (44.0)	2282 (43.0)
Hypertension, n (%)	9199 (86.8)	12,007 (91.7)	4742 (89.3)
Peripheral artery disease, n (%)	1006 (9.5)	737 (5.6)	138 (2.6)
Any prior myocardial infarction, n (%)	1285 (12.1)	1594 (12.2)	517 (9.7)
Any prior coronary artery bypass grafting, n (%)	612 (5.8)	1128 (8.6)	377 (7.1)
**Medications**			
Clopidogrel, n (%)	9990 (94.3)	12,462 (95.2)	5003 (94.2)
Ticagrelor, n (%)	393 (3.7)	447 (3.4)	210 (4.0)
Prasugrel, n (%)	674 (6.4)	663 (5.1)	310 (5.8)
Oral Anticoagulants, n (%)	538 (5.1)	973 (7.4)	346 (6.5)
Statins, n (%)	8226 (77.6)	10,662 (81.4)	4135 (77.9)
Discontinued DAPT < 10 months after PCI, n (%)	1429 (13.5)	1415 (10.8)	596 (11.2)
**Follow-up, Person-Months, mean (SD)**	68.4 (30.3)	69.5 (31.4)	70.0 (29.5)

DAPT=dual antiplatelet therapy, LDL=low density lipoprotein, PCI=percutaneous coronary intervention.

Table S3 shows the procedural characteristics at the index PCI including the stented arteries, the type of stent, stent dimensions and lengths, fluoroscopy time and contrast use. Compared to never and former smokers, current smokers were slightly more likely to have PCI to the right coronary artery, but had similar numbers of stents, fluoroscopy time, and contrast use.

**Fig. 2:**
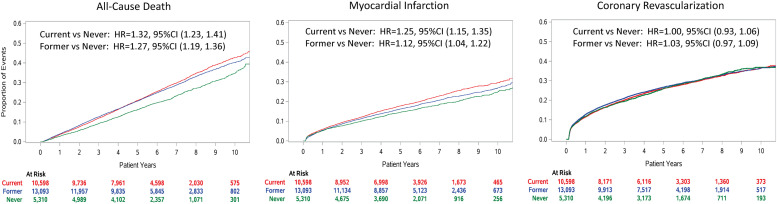
Event curves and hazard ratios (HR) and 95 % confidence intervals (95 %CI) from Cox proportional hazards models for all-cause death, myocardial infarction, and coronary revascularization after the index PCI.

By 10 years after the index PCI, 7896 (27.2 %) of subjects had died, 5349 (18.4 %) had a myocardial infarction, and 6592 (22.7 %) had repeat coronary revascularization. Compared to never smoking, current smoking associated with higher risks of all-cause death and myocardial infarction over the 10 years after PCI (Table 2 and Fig. 2). Former smoking also related to higher risks of all-cause death and myocardial infarction, but these were attenuated compared to current smoking. Repeat revascularization was not related to current or former smoking. Models restricted to subgroups of sex and presentation with or without an acute coronary syndrome at the index PCI gave similar results without significant interactions (Table S4). Competing risks models accounting for the competing risk of death also showed similar results for smoking status and risk of myocardial infarction or repeat coronary revascularization (Table S5).

**Table 2:** Hazard ratios and 95 % confidence intervals (95 % CI) for outcomes over follow-up by smoking status at baseline PCI.

Outcome		Current *n* = 10,598	Former *n* = 13,093	Never *n* = 5310
All-Cause Death	Number Events	3036	3679	1181
	Crude Hazard Ratio (95 %CI)	1.32 (1.23, 1.41)*	1.27 (1.19, 1.36)*	Reference
	Adjusted Hazard Ratio (95 %CI)	1.55 (1.44, 1.66)*	1.13 (1.06, 1.21)†	Reference
Myocardial Infarction	Number Events	2090	2390	869
	Crude Hazard Ratio (95 %CI)	1.25 (1.15, 1.35)*	1.12 (1.04, 1.22)	Reference
	Adjusted Hazard Ratio (95 %CI)	1.32 (1.21, 1.43)*	1.08 (1.00, 1.17)	Reference
Coronary Revascularization	Number Events	2836	3608	1448
	Crude Hazard Ratio (95 %CI)	1.00 (0.93, 1.06)	1.03 (0.97, 1.09)	Reference
	Adjusted Hazard Ratio (95 %CI)	0.98 (0.91, 1.04)	1.00 (0.94, 1.07)	Reference

⁎ *p* < 0.0001.

† *p* < 0.001 ^‡^*p* < 0.01 Covariates in adjusted models: age, sex, race, ethnicity, acute coronary syndrome at the index PCI, anemia, congestive heart failure, chronic kidney disease, insulin requiring diabetes mellitus, oral hypoglycemics, hypertension, peripheral artery disease, any prior myocardial infarction, any prior coronary artery bypass grafting, total cholesterol, low-density lipoprotein cholesterol, systolic blood pressure, body mass index, use of dual antiplatelet agents, anticoagulants, and statins.

Of the 7896 deaths, 6389 (81 %) had cause of death known. Over follow-up, there were 2755 (43 %) cardiovascular deaths, 1252 (20 %) cancer deaths, 518 (8 %) pulmonary deaths, 152 (2 %) other infection deaths, and 1712 (27 %) other causes of death. Compared to never smoking, current smoking related to higher risks of cardiovascular death, cancer death, pulmonary death, and other deaths (Table 3 and Figure S1). The risk of cardiovascular, cancer, and pulmonary death was higher with former smoking compared to never smoking, but with risks that were markedly attenuated compared to current smoking, consistent with a benefit from stopping smoking. Models including COPD gave similar results (Table S6), except for pulmonary deaths where COPD lies on causal pathway for this endpoint and is highly correlated with COPD death.

**Table 3:** Hazard Ratios and 95 % confidence intervals (95 % CI) for causes of death over follow-up by smoking status at baseline PCI.

Outcome		Current *n* = 9502	Former *n* = 12,009	Never *n* = 4860
Cardiovascular Death	Number Events	986	1344	425
	Crude Hazard Ratio (95 %CI)	1.20 (1.07, 1.34)‡	1.30 (1.17, 1.45)*	Reference
	Adjusted Hazard Ratio (95 %CI)	1.34 (1.19, 1.51)*	1.12 (1.00, 1.25)	Reference
Cancer Death	Number Events	563	545	144
	Crude Hazard Ratio (95 %CI)	2.02 (1.69, 2.43)*	1.56 (1.30, 1.87)*	Reference
	Adjusted Hazard Ratio (95 %CI)	2.55 (2.10, 3.08)*	1.41 (1.17, 1.70)†	Reference
Pulmonary Death	Number Events	255	226	37
	Crude Hazard Ratio (95 %CI)	3.57 (2.53, 5.04)*	2.51 (1.78, 3.56)*	Reference
	Adjusted Hazard Ratio (95 %CI)	4.07 (2..85, 5.83)*	2.06 (1.44, 2.93)*	Reference
Other Infection	Number Events	54	74	24
	Crude Hazard Ratio (95 %CI)	1.16 (0.72, 1.88)	1.27 (0.80, 2.01)	Reference
	Adjusted Hazard Ratio (95 %CI)	1.17 (0.70, 1.93)	1.05 (0.66, 1.67)	Reference
Other Death	Number Events	593	823	296
	Crude Hazard Ratio (95 %CI)	1.04 (0.90, 1.19)	1.14 (1.00, 1.31)	Reference
	Adjusted Hazard Ratio (95 %CI)	1.23 (1.06, 1.42)‡	1.01 (0.88, 1.16)	Reference

⁎ *p* < 0.0001.

† *p* < 0.001.

‡ *p* < 0.01.

The potential effect of smoking cessation on total events among the 10,958 subjects who smoked was estimated from the difference in absolute risks over 10 years between current versus former smoking (Table 4). The difference in risk indicates that smoking cessation could potentially decrease total deaths by 18.5 %, myocardial infarction by 14.8 %, cardiovascular deaths by 14.8 %, cancer deaths by 42.5 %, and pulmonary deaths by 48.3 %. The number of smokers needed to treat to prevent one event (NNT) would be 11.4 for all-cause deaths, 20.5 for myocardial infarction, 34.7 for cardiovascular deaths, 18.3 for cancer deaths, and 30.1 for pulmonary deaths.

**Table 4:** The impact of smoking cessation over 10 years estimated as the difference in risk between current smoking and former smoking (events prevented). The events prevented is expressed as a percentage of events in smokers after PCI (attributable risk in smokers) and the number of current smoking patients needed to stop smoking to prevent one event (number needed to treat).

Event	Risk of Event/ 100 subjects (95 % CI)		Events Prevented from Stopping Smoking/ 100 Smoking Patients (95 % CI)	Events Prevented as a% of All Events in Smokers (95 % CI)	Number Needed to Treat (95 % CI)
	Current Smoking	Former Smoking			
Death	47.6 (46.3, 48.8)	38.8 (37.7, 39.8)	8.8 (7.4, 10.2)	18.5 % (16.0 %, 20.9 %)	11.4 (9.8, 13.4)
Myocardial Infarction	33.1 (31.7, 34.5)	28.3 (27.1, 29.4)	4.9 (3.4, 6.4)	14.8 % (11.3 %, 18.0 %)	20.5 (15.7, 29.3)
Cardiovascular Death	19.4 (18.2, 20.6)	16.6 (15.6, 17.5)	2.9 (1.6, 4.2)	14.8 % (9.8 %, 19.6 %)	34.7 (24.0, 62.7)
Cancer Death	12.8 (11.6, 14.1)	7.4 (6.7, 8.1)	5.5 (4.2, 6.7)	42.6 % (37.7 %, 47.2 %)	18.3 (14.9, 23.8)
Pulmonary Death	6.8 (5.8, 7.9)	3.6 (3.0, 4.1)	3.3 (2.3, 4.3)	48.3 % (41.8 %, 54.2 %)	30.1 (23.0, 43.3)

95 % CI = 95 % confidence interval.

## 4 Discussion

Our study assessing the risk of adverse events over the decade after PCI with second-generation drug eluting stents and contemporary medical therapy showed that smoking associated with higher rates of death, myocardial infarction, cardiovascular death, cancer death, and pulmonary deaths. Former smoking associated with markedly attenuated risks for these events. The lower risk compared to current smoking highlights the potential benefits of stopping smoking as evident in the relatively low NNT. Thus, smoking continues to be a major reversible risk factor for adverse events after PCI in the modern era with improved stent designs and better guideline directed medical therapy.

Our analysis also highlights the high burden of non-cardiovascular deaths (accounting for over half the deaths) and particularly cancer and pulmonary deaths, which together account for 28 % of deaths. Our study illustrates the importance of preventing smoking and stopping smoking on cardiovascular *and* non-cardiovascular death at the individual patient level as reflected in the hazard ratios and NNTs.

In other studies, cardiovascular therapies such as lipid lowering and antithrombotic therapies have similar effects on cardiovascular outcomes as never smoking in this study [24,25]. However, our study shows that preventing smoking or smoking cessation are likely to have additional effects on the prevention of pulmonary and cancer deaths in patients after PCI. Other lifestyle therapies also have an impact beyond their importance in preventing cardiovascular disease. For, example the Mediterranean diet and other similar healthy food patterns likely also relate to lower risks of cancer and physical activity may prevent complications of lung disease [26,27].

The elevated risk of cancer and pulmonary deaths in smokers and former smokers also points to shared causes for these diseases and cardiovascular disease in general [26,28]. One example supporting this concept is the recent recognition of clonal hematopoiesis of indeterminate potential (CHIP), which is more prevalent in smokers and increased age. CHIP is a risk factor for cardiovascular disease, chronic obstructive pulmonary disease, and hematological cancers [29].

The Coronary Artery Surgery Study registry, published 4 decades ago before the evolution of PCI and many intensive medical treatments to reduce lipids and blood pressure, showed that men with coronary disease who stopped smoking had lower rates of death and myocardial infarction than those who continued to smoke [17]. Subsequent early reports of the relationship of smoking to cardiovascular disease after a cardiovascular event focused on shorter-term outcomes, and particularly the paradoxical lower risk of subsequent disease with current smoking patients presenting with stable angina or ACS and MI in the thrombolytic and PCI era [9,[30], [31], [32], [33]]. This paradox is largely explained by smokers having PCI at a younger age, less comorbidities, and more thrombotic lesions than non-smokers [9,11,14,34], an observation that is still evident today as shown in Table 1. Many studies don't distinguish former smoking from never smoking and this may confound the relationships with smoking [4,11,13]. Similar to our results, early studies using earlier PCI technologies suggest no increased risk or a lower risk of repeat coronary revascularization with current smoking. This may be due to a lower threshold for seeking medical help and subsequently lower rates of repeat angiography among current versus never smoking patients who have recurrent angina despite similar extent of coronary artery disease [13].

### 4.1 Limitations

Our study is an observational study and may not account for unknown confounders associated with smoking which may relate to the risk of adverse outcomes and could not be included in our extensive multivariable modeling. As a result, our estimate of the benefit from stopping smoking may be over- or under-estimated and should be considered hypothesis generating. Our study was from a Veteran population, the majority of whom are white and male, and had few women to reliably estimate the risks of smoking in women. The outcomes were based on ICD codes and on death certification, which may introduce a bias to increase the proportion of cardiovascular deaths compared to other causes of death. However, this would not diminish our conclusion that many deaths are from non-cardiovascular causes. We are not able to determine when former smoking patients discontinued smoking or if smoking status changed during follow-up. However, the risks for former smoking tended to lie between current and never smoking, suggesting our definition of former smoking indicated a status that had existed for some time before the index PCI. The NNT estimates for smoking cessation may not reflect the efficacy of smoking cessation as some patients who were classified as former smoking may have restarted smoking. However, our estimates may provide a more realistic estimate of the NNT for smoking cessation in actual practice as it would include some non-adherence to smoking cessation over follow-up.

### 4.2 Conclusions

In patients having contemporary medical therapy and PCI for coronary artery disease, we found current smoking related to higher risks of non-cardiovascular death in addition to previously reported increased risks of death, myocardial infarction, and cardiovascular death. These risks are attenuated by stopping smoking and analyses of absolute risk suggest large benefits from stopping smoking on cardiovascular and non-cardiovascular events (Central Illustration). Critical to this goal will be the development of new treatments for smoking cessation to improve the modest success from existing pharmacological and non-pharmacological methods [35]. The potential benefits of stopping smoking on non-cardiovascular events could be leveraged to encourage smoking cessation after PCI.

## CRediT authorship contribution statement

**Scott Kinlay:** Writing – review & editing, Writing – original draft, Supervision, Project administration, Methodology, Investigation, Conceptualization. **Melissa M. Young:** Writing – review & editing, Methodology, Investigation, Formal analysis, Data curation. **David R. Gagnon:** Writing – review & editing, Supervision, Methodology, Investigation, Conceptualization.

## Declaration of competing interest

The authors declare that they have no known competing financial interests or personal relationships that could have appeared to influence the work reported in this paper.

## Appendix Supplementary materials

Image, application 1
