# Supplementary material for: Smoking and 10-year risk of cardiovascular and non-cardiovascular events after contemporary coronary stenting
Source: Am J Prev Cardiol. 2024 Aug 15;19:100718. doi: 10.1016/j.ajpc.2024.100718 (PMC11380164; doi:10.1016/j.ajpc.2024.100718)

Supplementary Appendix

S. Kinlay, et al

Contents

[Table S1. ICD 9 and ICD 10 Codes for Comorbidities and Outcomes 2](#_Toc173225954)

[Table S2. Classification of Cause of Death 6](#_Toc173225955)

[Table S3. Description of Procedural Characteristics at the Time of the Index PCI 7](#_Toc173225956)

[Table S4. Risk by Subgroups of Sex and Acute Coronary Syndrome at the Index PCI 8](#_Toc173225957)

[Table S5. Analyses of Risk Accounting for Competing Risk of Death 9](#_Toc173225958)

[Table S6. Comparison of Models With and Without COPD as a Covariate 10](#_Toc173225959)

[Figure S1. Event curves for cause of death over 10 years 11](#_Toc173225960)

# Table S1. ICD 9 and ICD 10 Codes for Comorbidities and Outcomes

| CONDITION | ICD9-CM | ICD10-CM | ICD9-PCS | | | ICD10-PCS | CPT / HCPCS | |
| --- | --- | --- | --- | --- | --- | --- | --- | --- |
| Prior Percutaneous Coronary Intervention = prior drug-eluting stent (first or second-generation), bare metal stent, or balloon angioplasty | | | | | | | | |
| First-generation drug-eluting stent |  |  | | 36.07 | 0270346, 027034Z, 0720356, 027035Z, 0270366, 027036Z, 0270376, 027037Z | | | C1874, C1875 OR  [(G0290, G0291 before 2014)  OR  (C9600, C9601, C9602, C9603, C9604, C9605, C9606, C9607, C9608 from 2013 on)] |
| Second-generation drug-eluting stent |  |  | | 36.07 | 0270346, 027034Z, 0720356, 027035Z, 0270366, 027036Z, 0270376, 027037Z | | | C1874, C1875 OR  [(G0290, G0291 before 2014)  OR  (C9600, C9601, C9602, C9603, C9604, C9605, C9606, C9607, C9608 from 2013 on)] |
| Bare metal stent |  |  | | 36.06 | 02703D6, 02703DZ, 02703E6, 02703EZ, 02703F6, 02703FZ, 02703G6, 02703GZ | | | C1876, C1877 OR  [(92980 or 92981 before 2014)  OR  (92928, 92929, 92933, 92934, 92937, 92938, 92941, 92943, or 92944 from 2013 on)  AND no DES C codes] |
| PCI with balloon angioplasty |  |  | | 0.66 | 02703Z6, 02703ZZ | | | 92920, 92921 |
| Prior coronary artery bypass surgery (CABG) |  |  | | 36.1 | 02100A3, 02100A8, 02100A9, 02100AC, 02100AF, 02100AW, 0210093, 0210098, 0210099, 021009C, 021009F, 021009W | | | 33510-33516, 33517-33523, 33530, 33533-33536 |
| Stroke | 433.01, 433.11. 433.21, 433.31. 433.81, 433.91, 434.01, 434.11, 434.91, 436 | I63 | |  |  | | |  |
| Myocardial Infarction | 410 | I21 | |  |  | | |  |
| Major Bleed | 379.23, 423.0, 430, 431, 432, 568.81, 719.1 456.0, 459.0, 530.21, 530.7, 530.82, 531.00, 531.20, 531.40, 531.60, 531.01, 531.21, 531.41, 531.61, 532.00, 532.20, 532.40, 532.60, 532.01, 532.21, 532.41, 532.61, 533.00, 533.20, 533.40, 533.60, 533.01, 533.21, 533.41, 533.61, 534.00, 534.20, 534.40, 534.60, 534.01, 534.21, 534.41, 534.61, 535.01, 535.11, 535.21, 535.31, 535.41, 535.51, 535.61, 535.71, 537.83, 562.12, 562.13, 569.3, 569.85, 578.0, 578.1, 578.9, 784.8, 786.3 | H43.1, I31.2, I60, I61, I62, K66.1, M25.0, K22.11, K22.6 , K25.0, K25.2, K25.4, K25.6, K26.0, K26.2, K26.4, K26.6, K27.0, K27.2, K27.4, K27.6, K28.0, K28.2, K28.4, K28.6, K29.01, K29.21, K29.31, K29.41, K29.51, K29.61, K29.71, K29.81, K29.91, K31.811, K55.21, K57.01, K57.13, K57.21, K57.31, K57.33, K57.41, K57.51, K57.53, K57.81, K57.91, K57.93, K62.5, K92.1, K92.2, R04.1, R04.8, R04.89, R04.9 | |  |  | | |  |
| Chronic kidney disease | 585 | N18 | |  |  | | |  |
| Diabetes | 250 | E08, E09, E10, E11, E13 | |  |  | | |  |
| Peripheral Artery Disease | 440.2 | I70.2, I70.3, I70.4, I70.5, I70.6, I70.6, I70.92 | |  |  | | |  |
| Hypertension | 401 | I10, I15, I16 | |  |  | | |  |
| Chronic Obstructive Pulmonary Disease (COPD) | 490, 491, 492, 494, 496 | J41, J42, J43, J44 | |  |  | | |  |
| Congestive Heart Failure | 428 | I50 | |  |  | | |  |
| Angina | 413 | I20.1, I20.8, I20.9, I25.11, I25.70, I2571, I25.72, I25.73, I25.75, I25.76, I25.79 | |  |  | | |  |
| Cancer | 140 - 209.3, 209.7 | C00-C26, C30-C34, C37-C41, C43-C58, C60-C85, C7A, C88, C90-C96, D45 | |  |  | | |  |
| Chemotherapy | V58.1 | Z51.11, Z51.12 | |  |  | | | 964, 96535, 96538, GA0498, J9, Q0083, Q0084, Q0085, 4180F, 9955, C8953, C8954, C8955, G0070, G0355, G0359, G0361, G8372, G8373, G9829, J7150, Q0083, Q0084, Q0085, S9329, S9330, Z0904 |
| Radiotherapy | V58.0 | Z51.0, | |  |  | | | 77371, 77372, 77373, 77381, 77385, 77386, 7740, 77410, 77411, 77412, 77413, 77414, 77416, 77417, 77418, 77420, 77422, 77423 ,77424, 77425, 77427, 7743, 7746, 77499, 775, 77761, 77762, 77763, 77771, 77772, 77773, G0174, G0256, G0261, G6003, G6004, G6005, G6006, G6007, G6008, G6009, G6010, G6011, G6012, G6013, G6014, G6015, G6016, 0082T, 4165F, 4818F, 4812F, Z0903 |
| Anemia | 281, 283, 284, 285 | D51, D52, D53, D59, D60, D61, D62, D63, D64 | |  |  | | |  |
| Acute Coronary Syndrome | 410, 411.1, 411.8 | I21, I20.0 | |  |  | | |  |

# Table S2. Classification of Cause of Death

| **Classification** | **ICD 10 Codes** | **ICD10 Description** |
| --- | --- | --- |
| Cardiovascular Death | I26-I28 | Pulmonary heart disease and diseases of pulmonary circulation |
|  | I60-I69 | Cerebrovascular Diseases |
|  | I70-I79 | Diseases of arteries, arterioles and capillaries |
|  | I80-89 | Diseases of veins, lymphatic vessels and lymph nodes, not elsewhere classified |
|  | I20-I25 | Ischemic heart diseases |
|  | I30-I52 | Other forms of heart disease |
|  | R99 | Unknown |
| Cancer Death | C00-C97 | Malignant Neoplasms |
| COPD or Pulmonary Infection Death | J00-J06 | Acute upper respiratory infections |
|  | J09-J18 | Influenza and Pneumonia |
|  | J20-J22 | Other acute lower respiratory infections |
|  | J40-J47 | Chronic lower respiratory diseases |
| Other Infection Death | A00-A99 | Infections |
|  | B00-B99 | Other specific infections |
| Other Cause of Death | Everything not defined above |  |

# Table S3. Description of Procedural Characteristics at the Time of the Index PCI

| **Baseline Characteristics** | **Baseline Smoking Status** | | |
| --- | --- | --- | --- |
|  | **Current n=10,598** | **Former n=13,093** | **Never n=5,310** |
| Target Coronary Artery |  |  |  |
| Left anterior descending, n (%) | 4,237 (40.1) | 5,434 (41.6) | 2,504 (47.3) |
| Left circumflex, n (%) | 3,234 (30.6) | 3,740 (28.6) | 1,506 (28.5) |
| Right coronary, n (%) | 3,550 (33.6) | 4,067 (31.1) | 1,512 (28.6) |
| Left main, n (%) | 318 (3.0) | 496 (3.8) | 130 (2.5) |
| Graft (arterial or venous), n (%) | 520 (4.9) | 923 (7.1) | 295 (5.6) |
|  |  |  |  |
| Stent type |  |  |  |
| Endeavor, n (%) | 575 (5.4) | 686 (5.2) | 250 (4.7) |
| Promus, n (%) | 2,789 (26.3) | 3,378 (25.8) | 1,313 (24.7) |
| Resolute, n (%) | 1,725 (16.3) | 2,221 (17.0) | 982 (18.5) |
| Xience, n (%) | 5,941 (56.1) | 7,328 (56.0) | 2,977 (56.1) |
|  |  |  |  |
| Number of stents |  |  |  |
| 1 stent, n (%) | 6,532 (62.3) | 8,086 (62.3) | 3,259 (61.9) |
| 2 stents, n (%) | 2,625 (25.0) | 3,337 (25.7) | 1,347 (25.6) |
| 3 or more stents, n (%) | 1,336 (12.7) | 1,560 (12.0) | 657 (12.5) |
| Number of stents, mean (SD) | 1.50 (0.7) | 1.50 (0.7) | 1.51 (0.7) |
|  |  |  |  |
| Stent Dimensions |  |  |  |
| Minimum diameter mm, mean (SD) | 2.87 (0.5) | 2.86 (0.4) | 2.85 (0.5) |
| Maximum diameter mm, mean (SD) | 3.03 (0.5) | 3.01 (0.5) | 3.00 (0.5) |
| Total Stent length mm, mean (SD) | 31.7 (22.0) | 31.2 (21.3) | 31.0 (21.0) |
|  |  |  |  |
| Fluoroscopy time index PCI min, mean (SD) | 18.3 (198.3) | 17.6 (74.1) | 20.2 (150.2) |
| Total contrast volume index PCI mL, mean (SD) | 205.3 (617.8) | 199.0 (102.9) | 199.6 (113.3) |

# Table S4. Risk by Subgroups of Sex and Acute Coronary Syndrome at the Index PCI

Hazard ratios adjusted for: Age, Sex, Race, Ethnicity, Anemia, Congestive Heart Failure, Chonic Kidney Disease, Diabetes Mellitus, Hypertension, Peripheral Artery Disease, Prior Myocardial Infarction, Prior Coronary Artery Bypass Grafting, Oral Anticoagulation, Statins, Total Cholesterol, LDL Cholesterol, Body Mass Index, Systolic Blood Pressure

**S4A. Subgroups of men and women. Cause of death omitted due to too few numbers of events in women (<11 events per cause of death.**

**S4B. Subgroups of acute coronary syndrome at the index PCI.**

# Table S5. Analyses of Risk Accounting for Competing Risk of Death

Hazard ratios and 95% confidence intervals from competing risks models. For comparison the hazard ratios from cause-specific models from Cox Proportional Hazards are also shown.

|  |  | **Adjusted Risk Competing Risks** | | **Adjusted Risk Cause-Specific** |
| --- | --- | --- | --- | --- |
| **Outcome** | **Baseline Smoking** | **HR (95% CI)** | **P-value** | **HR (95% CI)** |
| Myocardial Infarction | Current | 1.27 (1.17,1.38) | <.0001 | 1.32 (1.21, 1.43) |
|  | Former | 1.08 (0.99,1.17) | 0.07 | 1.08 (1.00, 1.17) |
|  | Never | REF |  | REF |
| Repeat Coronary Revascularization | Current | 0.96 (0.90,1.03) | 0.2850 | 0.98 (0.91, 1.04) |
|  | Former | 0.99 (0.93,1.06) | 0.8022 | 1.00 (0.94, 1.07) |
|  | Never | REF |  | REF |

# Table S6. Comparison of Models With and Without COPD as a Covariate

|  |  | **Multivariate** | | **Multivariate + COPD** | |
| --- | --- | --- | --- | --- | --- |
| **Outcome** | **Baseline Smoking** | **HR (95% CI)** | **P-value** | **HR (95% CI)** | **P-value** |
| Death | Current | 1.62 (1.51,1.74) | <.0001 | 1.45 (1.35,1.56) | <.0001 |
|  | Former | 1.15 (1.07,1.23) | <.0001 | 1.09 (1.02,1.16) | 0.0164 |
|  | Never | REF |  |  |  |
| Myocardial Infarction | Current | 1.34 (1.23,1.46) | <.0001 | 1.29 (1.18,1.41) | <.0001 |
|  | Former | 1.08 (1.00,1.17) | 0.0570 | 1.06 (0.98,1.15) | 0.1392 |
|  | Never | REF |  | REF |  |
| Revascularization | Current | 0.96 (0.90,1.03) | 0.2850 | 0.98 (0.91,1.05) | 0.5130 |
|  | Former | 0.99 (0.93,1.06) | 0.8022 | 1.00 (0.94,1.06) | 0.9564 |
|  | Never | REF |  |  |  |
| Cardiovascular Death | Current | 1.40 (1.24,1.58) | <.0001 | 1.30 (1.15,1.47) | <.0001 |
|  | Former | 1.14 (1.02,1.27) | 0.0236 | 1.10 (0.98,1.23) | 0.1028 |
|  | Never | REF |  | REF |  |
| Cancer Death | Current | 2.60 (2.14,3.14) | <.0001 | 2.21 (1.82,2.69) | <.0001 |
|  | Former | 1.41 (1.17,1.70) | 0.0003 | 1.31 (1.09,1.58) | 0.0046 |
|  | Never | REF |  | REF |  |
| COPD /Pulmonary Infection Death | Current | 4.38 (3.06,6.28) | <.0001 | 2.52 (1.75,3.64) | <.0001 |
|  | Former | 2.13 (1.49,3.03) | <.0001 | 1.57 (1.10,2.25) | 0.0131 |
|  | Never | REF |  | REF |  |
| Other Infection Death | Current | 1.29 (0.78,2.14) | 0.3169 | 1.22 (0.73,2.05) | 0.4470 |
|  | Former | 1.07 (0.67,1.71) | 0.7784 | 1.04 (0.65,1.67) | 0.8701 |
|  | Never | REF |  | REF |  |
| Other Death | Current | 1.28 (1.10,1.48) | 0.0010 | 1.21 (1.04,1.41) | 0.0127 |
|  | Former | 1.02 (0.89,1.17) | 0.7649 | 0.99 (0.87,1.14) | 0.9276 |
|  | Never | REF |  | REF |  |

# Figure S1. Event curves for cause of death over 10 years


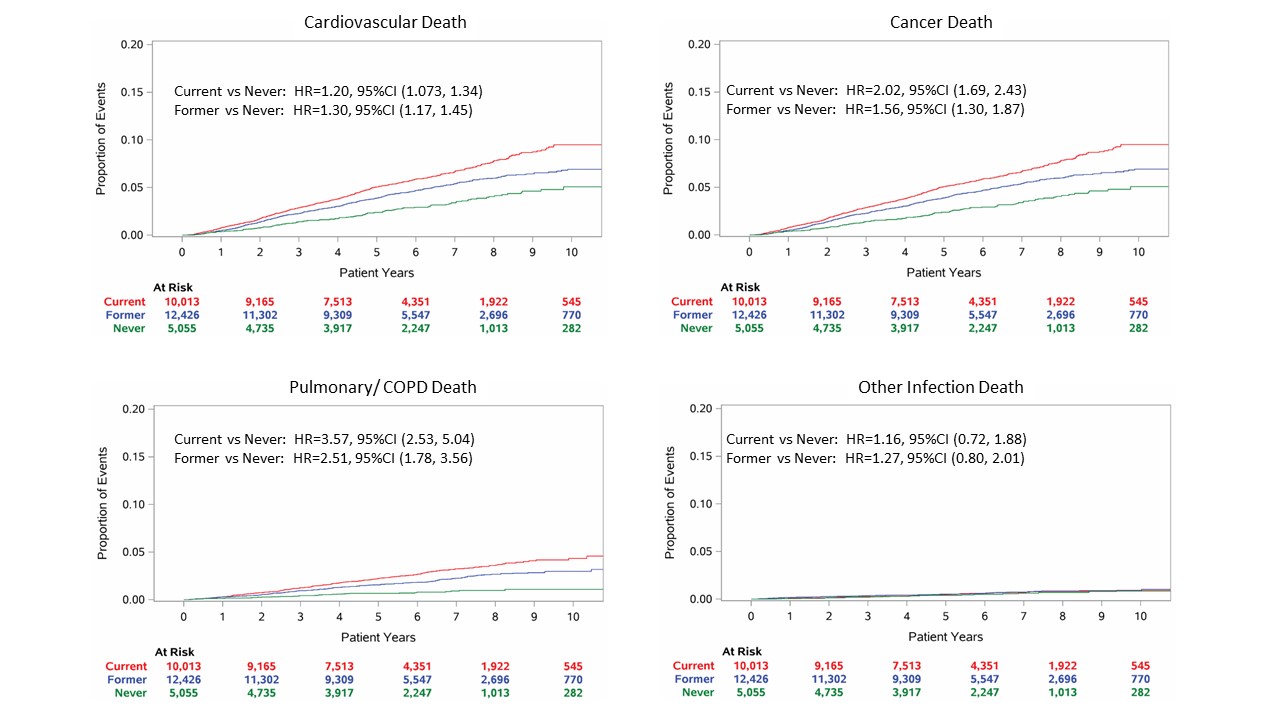

Supplement: Supplementary file 1 [file mmc1.docx]
